# Manipulating rod-shaped bacteria with optical tweezers

**DOI:** 10.1038/s41598-019-55657-y

**Published:** 2019-12-13

**Authors:** Zheng Zhang, Tom E. P. Kimkes, Matthias Heinemann

**Affiliations:** Molecular Systems Biology, Groningen Biomolecular Sciences and Biotechnology Institute, University of Groningen, Nijenborgh 4, 9747 AG Groningen, the Netherlands

**Keywords:** Optical tweezers, Escherichia coli, Wide-field fluorescence microscopy

## Abstract

Optical tweezers have great potential in microbiology for holding and manipulating single cells under a microscope. However, the methodology to use optical tweezers for live cell studies is still at its infancy. In this work, we determined suitable parameters for stable trapping of single *Escherichia coli* bacteria, and identified the upper limits of IR-exposure that can be applied without affecting viability. We found that the maximum tolerable IR-exposure is 2.5-fold higher when employing oscillating instead of stationary optical trapping (20 J and 8 J, respectively). We found that good stability of cells in an oscillating trap is achieved when the effective trap length is 20% larger than the cell length, the oscillation frequency higher than 100 Hz and the trap oriented perpendicular to the medium flow direction. Further, we show, using an IR power just sufficient for stable holding, that bacteria remain viable during at least 30 min of holding in an oscillating trap. In this work, we established a method for long-term stable handling of single *E. coli* cells using optical tweezers. This work will pave the way for future use of optical tweezers in microbiology.

## Introduction

In the past decade, we witnessed a transition in microbiology from bulk to single-cell studies, driven by exciting developments in microfluidics and time-lapse microscopy. Microfluidic devices now enable precise control of the micro-environment of single cells in long-term studies and advanced microscopic techniques greatly expanded spatial and temporal resolution, allowing dynamic and quantitative measurements on single cells^1–5^. Now being able to culture and visualize single cells, the next challenge is to handle and manipulate them, to study for instance the response of individual cells upon cell-to-cell interactions, or contact with surfaces.

Here, optical tweezers (OT) offer great potential. Traditionally, optical tweezers have been used for single-molecule studies, such as the interaction between DNA and repair complexes or helicases^6–8^. OT were also applied in microbiological studies, such as cell sorting in microfluidics, investigating cellular responses upon rapid switching of cells between different media^9,10^, or pulling apart cell clusters in biofilm studies^11^. Also, optical trapping of single bacteria allowed the observation of flagellar motion with fluorescence microscopy to characterize the change of cell motion^12–14^ or to indicate cell viability upon environmental perturbations^15^, and was used to study cellular responses to inhibition of flagellar rotation^16^.

However, before OT can be widely applied to microbiology, there are still a number of issues that need to be solved. For instance, rod-shaped bacteria, such as *E. coli*, will orient along the beam axis of the optical trap, leaving only the short cross section of the cells to be visible during microscopic observation. To allow for the visualization of the long axis of the cell, apart from applying techniques like dual-beam or holographic trapping to the setup^11,13,17^, a single optical beam can simply be moved with high frequency along a distance comparable to the cell length, which will create an effective linear trap with a length twice the oscillation amplitude, forcing the cell to align along the focal plane^18–21^. However, such oscillating trapping of rod-shaped bacteria can be achieved by a range of settings (for instance, for the scanning frequency and trap length), and eventual effects of these settings on stability of holding and viability of trapped bacteria are yet to be investigated.

An additional issue when applying OT in biological studies is that the infrared (IR) light typically used for optical trapping, can induce cell damage. While the IR damage has been investigated in bacteria, the reported lethal dose of IR-exposure to optically trapped *E. coli* varied widely in different studies^22–25^, being as low as 0.54J in one report^24^, while another found no IR-induced damage at all^22^. Therefore, it is still unresolved how long a bacterial cell can actually be held by OT before damage occurs, and whether an oscillating trap could ameliorate this damage to some extent. Essentially, it is unclear what the optimal way of trapping is for rod-shaped bacteria, and what the limits are for the use of OT in microbiological research.

Here, towards establishing optical tweezers as a research tool to manipulate single live bacteria, we compared different trapping settings and investigated the upper limits of IR-exposure that can be applied without affecting viability. We found that cells could be trapped with an oscillating 1064 nm laser trap with a maximal IR dose of 20 J without compromising cell growth and gene expression, which is 2.5-fold higher than in a stationary trap. Using a laser intensity that is just sufficient for stable trapping, bacteria could be held in an oscillating trap for at least 30 min without compromising viability. Further, we identified settings for oscillating trapping (trap length, frequency and orientation of the trapped cell with regard to the flow direction of the medium) that provide a good stability of optically trapped cells. Our work demonstrates that, with carefully chosen settings, oscillating trapping provides a stable, low-damage method for manipulating single living *E. coli* cells and is, therefore, an effective tool for future single-cell bacterial studies.

## Results

### Microfluidic setup

**Figure 1:**
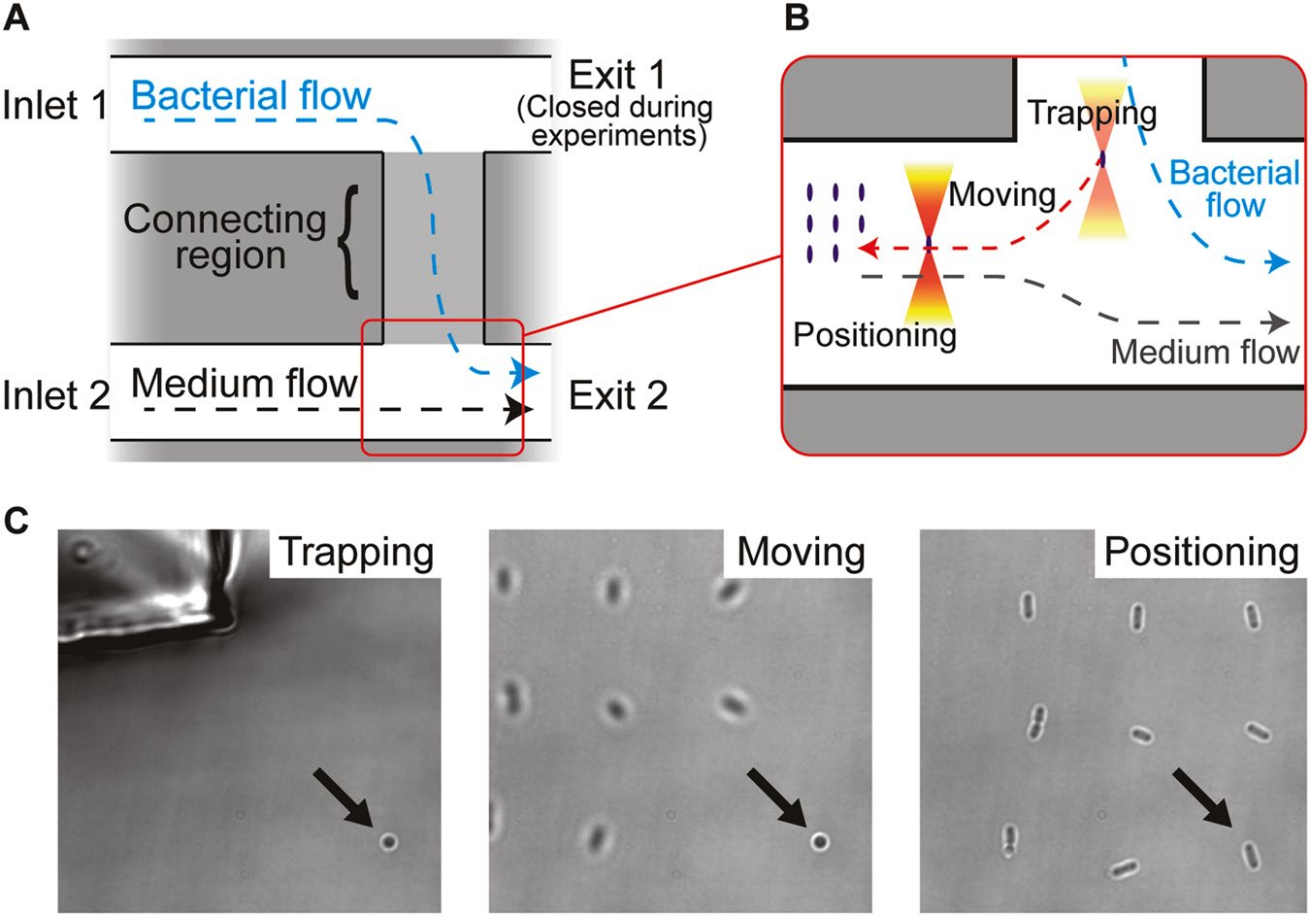
Microfluidic chip and optical trapping procedure. (**A**) Layout of the microfluidic chip, with indicated flow directions of medium and bacterial culture. (**B**) In all experiments, the procedure commenced with optical trapping of single *E. coli* bacteria near the connecting region and moving the trapped cells into the channel with flow of fresh medium. In experiments testing the survivability after optical trapping, cells were then held until they had been exposed to a predefined dose of IR light and thereafter positioned on the silanised cover glass. When testing the stability of the optical trapping, the flow rate in the medium channel was gradually increased and the flow rate at which the cell was lost from the trap was recorded. (**C**) Bright field images corresponding to the steps shown in (**B**). Here, a bacterium (indicated with an arrow) held with a stationary trap was moved into the medium channel. Just before positioning, oscillation of the trap was initiated to align the cell along the focal plane. Then, the trap was moved down to the surface of the silanised cover glass, so that the trapped cell was positioned on the cover glass with the long cell axis visible for imaging.

To facilitate optical trapping and long-term observation of *E. coli*, a microfluidic setup was used in combination with an air-pressurized system to control the flow of medium. The microfluidic chip contained two channels, which were connected by a shallow channel in between (Fig. 1A). A dilute *E. coli* culture and glucose minimal medium supplemented with IPTG were perfused into the two channels, respectively. Because the outlet of the channel with bacteria (‘Exit 1’, Fig. 1A) was closed during the experiment, the bacteria flowed through the shallow connecting region.

Single cells carrying an IPTG-inducible plasmid encoding GFP were trapped with the OT at the connection region (Fig. 1B). After exposure to a predefined dose of IR light (i.e. determined by laser intensity and holding time), cells were positioned onto the cover glass in the channel with flow of medium containing IPTG, where they were immobilized on a coating of (3-aminopropyl)triethoxysilane (APTES). Thereafter, their growth and GFP fluorescence were observed through time-lapse imaging. With this setup, we were able to optically trap single *E. coli* cells, expose them to a defined dose of IR light, immobilize them and observe cell growth and fluorescent protein production by time-lapse microscopy with constant supply of fresh medium.

### Stable trapping with reduced photodamage is achieved by oscillating trapping

**Figure 2:**
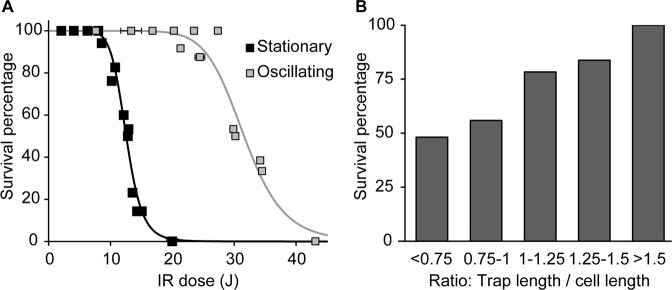
Effect of optical trapping on viability. (**A**) Survival percentage of *E. coli* after being trapped with stationary and oscillating optical traps with various IR doses. For oscillating traps, trap length and frequency were 3 μm and 330 Hz, respectively. The IR dose is the product of laser power and holding time. In the experiments, the laser power was varied from 56 to 72 mW, and the holding time from less than one minute to ten minutes. Each symbol was determined from at least 7 cells (average: 13 cells). Data are from 12 experiments. Lines are for illustration purposes. (**B**) Increasing the ratio of trap to cell length in the oscillating trap method leads to a higher survival percentage. Cells were exposed to 30 J of IR dose in an oscillating trap with 280 Hz of scanning frequency and 2.5 to 4.5 μm trap length. For every trapped cell, its length was determined according to a bright field picture taken immediately after the positioning step. Each bin contains at least 27 cells. If the laser intensity was increased for larger trap lengths, to keep a constant flux density, a decrease in survival was observed (Supplementary Figure S2A).

To quantify the maximum IR dose that can be used without affecting the viability of *E. coli* in stationary and oscillating traps, cells were exposed to different doses of IR light, after which growth and protein synthesis were recorded as indications of viability. IR doses are defined as laser power multiplied with holding times. To modulate the IR doses, we varied the laser powers from 56 to 72 mW and the holding times from less than one minute to ten minutes. With both stationary and oscillating traps, as expected, we found a negative correlation between IR dose and survival (Fig. 2A). While the first non-growing cells without fluorescent protein production were observed at IR doses of 8.6 J with the stationary trap, this happened at 21.3 J with the oscillating trap. Increasing the IR dose beyond these values rapidly reduced cell survival. In surviving cells, optical trapping did not have a significant effect on the growth rate and GFP production (Supplementary Figure S1). Thus, the maximum IR dose that can be used for trapping *E. coli* without affecting survival was determined to be 8 J for stationary trapping and 20 J for oscillating trapping. This finding implied that, with the same IR intensity, oscillating traps exert less damage and could be a less-invasive option for trapping cells. As the IR dose is the product of laser power and holding time, the maximally possible holding time without cell damage is determined by the laser power that is needed for stable cell trapping.

To investigate whether the reduced photodamage of oscillating trapping was because the whole cell body was not constantly irradiated during trapping, several trap lengths were tested. We expected to see less cell damage from oscillating traps when the trap length was longer than the cell length, as the beam would irradiate on the area outside of the cell for a fraction of time. To test this hypothesis, cells were exposed to 30 J in oscillating traps with various scanning lengths. Here, we found that the survival percentage increased almost linearly with the ratio between trap length and cell length (l_trap_/l_cell_) (Fig. 2B), increasing about 2-fold within the tested range of l_trap_/l_cell_. In line with our hypothesis, cells were less damaged by optical trapping when the relative trap length was longer. In cases where the trap oscillated over a length shorter than the bacterium (i.e. l_trap_/l_cell_ < 1) we noticed that the cells could no longer be maintained parallel to the focal plane but were tilted in the trap. This observation has been reported before^15^ and provides a way to image rod-shaped bacteria from any desired direction, for example for three-dimensional observation of localized fluorescence.

To test the effect of cell orientation on survival we trapped cells with two stationary beams, so that the orientation could be controlled. Specifically, when both IR beams were focused on the same position, effectively there would be one stationary beam and thus the cell would orient along the optical axis. In contrast, if the two beams were focused on the cell poles, the cell could be manipulated to assume an orientation parallel to the focal plane. We found that in the latter case, survival was substantially increased (Supplementary Figure S2B), indicating that less photodamage is caused to the cell when not oriented along the optical axis, presumably because less light passes through the cell.

### Most stable oscillating trap is achieved when trap length is 20% longer than cell length

Although the oscillating trapping method could reduce IR damage compared to stationary optical trapping of bacteria, the factors that determine how stable the cells can be held in a flow of medium with the former trapping method are still to be uncovered. To identify these factors, we measured the maximal flow rate at which cells can be retained in the oscillating trap operated with various trap lengths and oscillation frequencies.

**Figure 3:**
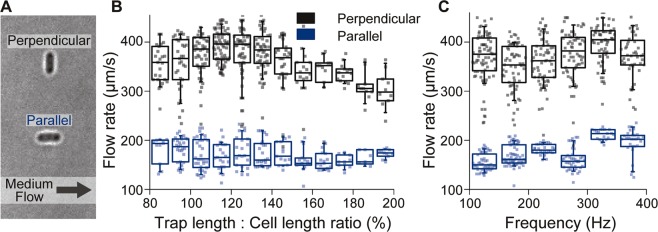
Factors that affect the stability of oscillating trapping. (**A**) Two cells in oscillating traps, which were set either perpendicular to or parallel with the flow direction of the medium. (**B**) Minimum flow rate that pushes cells out of the oscillating trap versus the ratio of trap to cell length. *E. coli* trapped by an oscillating trap were oriented perpendicular (black, n = 412) or parallel (blue, n = 228) to the flow direction. The trapping power was 72 mW, while the oscillating trap length and frequency ranged from 2.5 to 5 μm and 100 to 400 Hz, respectively. Quantile and median are shown as boxes, with the whiskers indicating 10–90% percentile. Data are from 6 experiments. (**C**) Same data as in B, but plotted against the frequency of the trapping oscillations.

Our first aim was to determine which orientation of the cell (i.e. perpendicular or parallel; Fig. 3A) with regard to the flow direction of the medium is more stable. Here, we found that the stability was more than two-fold higher if the cell was oriented perpendicular to the flow direction, compared to the parallel orientation (Fig. 3B). Towards explaining this finding, we first ruled out technical reasons (e.g. aberrations in the optics or scanning of the galvanometric mirrors) by testing the stability of both orientations in a microfluidic chip that was rotated by 90°, where we again found the perpendicularly oriented cells to be more stable (Supplementary Figure S3A). Second, we then held cells with two stationary traps (one on each end), instead of one oscillating beam, in both orientations. Here, we found that they were equally stable (Supplementary Figure S3B). This suggested that the movement of the optical trap in the same direction as the drag force creates a momentum that, at high flow rates, cannot be fully compensated when the trap moves back against the flow, thereby leading to a less stable situation in the parallel orientation.

We next asked if the ratio of trap to cell length would affect the stability of trapping. We thus determined this l_trap_/l_cell_ ratio for each cell. Here, we found that the most stable trap was obtained when the trap length was around 20% larger than the cell length (Fig. 3B). When the orientation of the scanning trap was parallel to the medium flow direction, no obvious trend could be observed between trapping stability and length ratio. Notably, the scanning frequency did not affect trapping stability (Fig. 3C). However, with frequencies below 100 Hz, the oscillating trap could not be established as the long axis of the cell would remain perpendicular to the focal plane and the cell would stay at one end of the linear trap, as previously reported^18^. On the other hand, high oscillation frequency did negatively affect survival (Supplementary Figure S2C), so ideally the frequency should not be set much higher than 100 Hz. Overall, maximal trapping stability of the oscillating method could be achieved if the cell orientation is set perpendicular to the medium flow direction, with a trap length 20% longer than the trapped cell and at a frequency higher than 100 Hz.

To test whether also other rod-shaped microorganisms can be trapped with these settings, we used *B. subtilis* and tested the abovementioned l_trap_/l_cell_ ratio. Here, we found that > 5-fold higher laser powers were needed in order to hold the cell stably. A higher power might be needed for *B. subtilis* because, unlike *E. coli*, *B. subtilis* is a gram-positive microorganism, meaning that it has cell wall differences compared to the gram-negative *E. coli*, which might yield different optical properties and thus exhibiting different trapping behaviour.

### *E. coli* can remain viable after 30 min of optical trapping

Oscillating trapping exerts less photodamage to *E. coli* than stationary trapping (Fig. 2A), while still allowing for stable holding with carefully chosen oscillation settings (Fig. 3). At the same time, oscillating traps offer the benefit of holding bacteria in the required orientation for imaging. However, even with the reduced photodamage, bacteria could only be held for 5–6 minutes before the first nonviable bacteria were observed (Fig. 2A), which would be a severe limitation for the application of OT in microbiological studies.

To investigate whether a lower IR laser power would allow us to handle single bacteria for a prolonged time, we first determined the minimum power required for stable holding. By holding cells in a microfluidic channel with 0.5 μL/min flow rate (corresponding to a velocity of ~65 μm/s) and gradually lowering the laser power until the cell was lost from the oscillating optical trap (l_trap_/l_cell_ = 1.2; perpendicular to flow direction), we established that at powers below 7.2 ± 2.0 mW it was no longer possible to stably hold an *E. coli* cell (Supplementary Figure S4).

**Figure 4:**
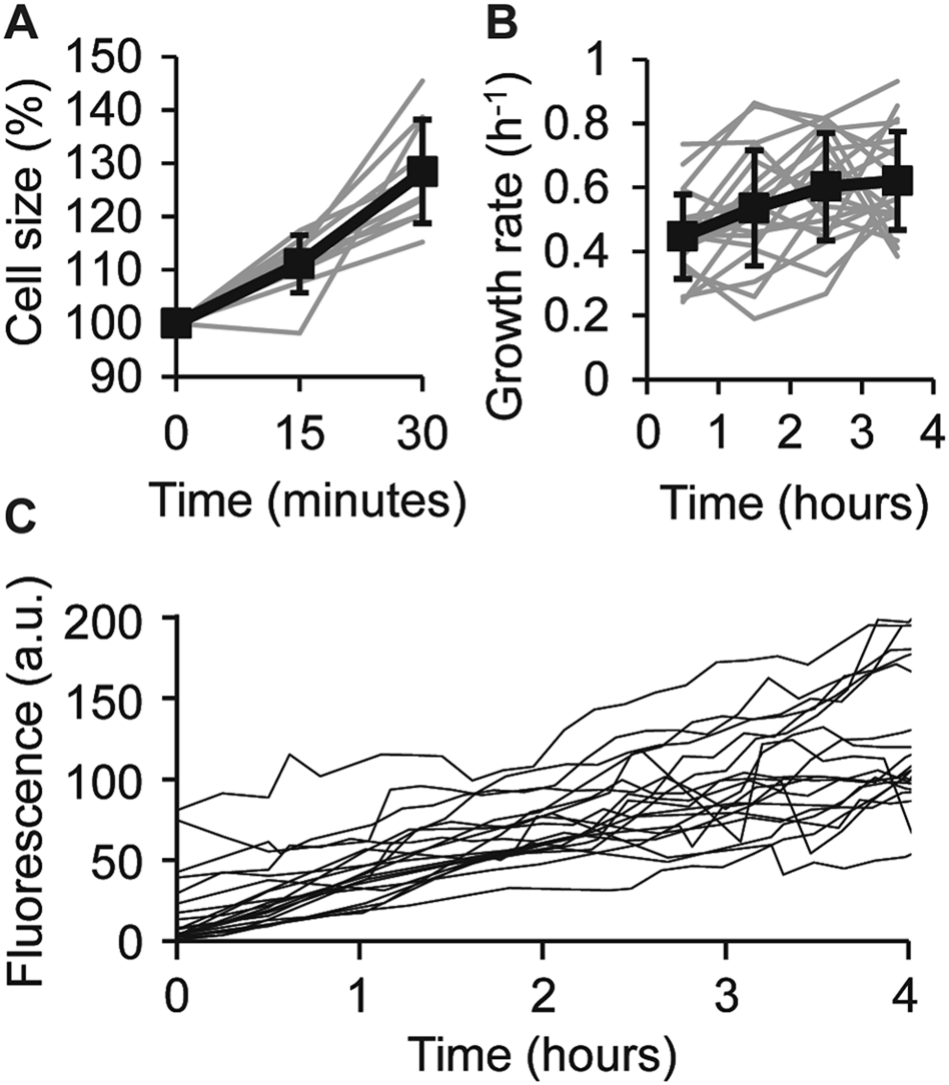
Growth and fluorescence of bacteria optically trapped for 30 min. Bacteria were held in a 0.25 μl/min medium flow for 30 min in an oscillating optical trap (l_trap_/l_cell_ = 1.3 at t = 0; oriented perpendicular to the flow) of 8 mW IR power, positioned on the cover glass and followed in time-lapse. (**A**) Relative cell size (normalized to the first time point) while holding; n = 10. (**B**) Growth rate in each hour after optical trapping. The first hour includes the 30 min holding; n = 20. (**C**) GFP fluorescence intensities in the first 4 h after optical trapping. From time point 0 until 0.5 h, the bacteria were held by the OT; n = 20.

To investigate survival upon prolonged IR exposure with this minimally necessary laser power, single cells were held for 30 min with an IR intensity of 8 mW. A higher IR power (40 mW) was shortly used (around 30 seconds) to initially trap the cells, to keep them more stable while taking an image after 15 min and when positioning them on the cover glass after the 30 min. We found that all bacteria survived the 30 min optical trapping (IR dose: ~18 J), which is consistent with the survival curve presented in Fig. 2A. While holding, the cells continued growing (Fig. 4A), albeit at a lower than normal growth rate: 0.48 ± 0.16 h^−1^. In the ensuing hours, the growth rate recovered almost fully, reaching 0.62 ± 0.15 h^−1^ (Fig. 4B). All cells that were held for 30 min expressed IPTG-inducible GFP (Fig. 4C).

## Discussion

In this work, we identified operational procedures that allow handling and imaging of single *E. coli* cells with optical tweezers, while minimizing negative effects on cell growth and protein synthesis. Specifically, by observing cell growth and fluorescent protein production after IR exposure, we investigated the correlation between the dose of IR-exposure and cell survival percentage when *E. coli* were held in two types of optical traps. We found that *E. coli* can sustain an IR dose of 8 J in stationary traps, while up to 20 J is tolerated in oscillating optical traps (Fig. 2). Further, we found that the stability of oscillating trapping is improved when the orientation of the trap is perpendicular to the medium flow and when the ratio of trap length to cell length is 120% (Fig. 3). Using a laser power just sufficient for stable holding, bacteria can be held for at least 30 min without compromising viability (Fig. 4).

The correlation of *E. coli*’s survival percentage with IR dose measured in this work is in accordance with Ref. ^25^, although wavelengths ranging from 840 to 930 nm were used there for trapping. When comparing our results to an earlier work, where the same IR wavelength was used^24^, we found that the maximum IR dose that can be used without affecting viability, was much higher in our experiments. Possibly, the difference between these studies is related to an increase in temperature, because in the previous study cells were trapped in a chamber without medium flow. Generally, it is considered that 100 mW of 1064 nm light causes a 1 °C rise in temperature, although larger temperature increases have also been reported^26^. In our study, however, we expect heating to play a negligible role in cell damage, as we used a lower IR intensity, and there was a flow of medium that should reduce heating of the trapped cell. While the thermal effect from trapping could possibly have an influence on cell survival, as supported by findings in *E. coli* and yeast^27,28^, the role of heating in OT-induced cell damage is ill-defined: Results obtained under anaerobic conditions suggest that rather generation of reactive oxygen species (ROS) is the major contributor to cell damage, as considerably less damage was caused under anaerobic compared to aerobic conditions^23^.

Oscillating trapping to manipulate the orientation of rod-shaped bacteria has previously been presented and has been used for Z-ring studies^18–21^. However, as a possible approach for holding cells in a microfluidic setup with flow of medium, the photodamage that oscillating trapping exerts to cells had not been investigated. We found that the photodamage caused by oscillating trapping is significantly lower compared to stationary trapping. Our results suggest that the decreased damage is partly due to the beam not focussing on the cell part of the time, as higher l_trap_/l_cell_ ratios give a higher chance for survival of the cell. This would be in line with the findings of increased cell viability in time-sharing traps, where one beam repeatedly scans through an array of predefined positions and stays at each position just long enough to keep the cell trapped^25^. However, this may not be the full explanation, as survival is still higher in an oscillating beam with l_trap_/l_cell_ ratio below 1, than in a stationary trap, while in both cases the cell is constantly exposed to the IR light. We conjecture that damage is higher in a stationary trap due to the fact that light beams are commonly less strongly focussed in the longitudinal direction, such that the whole cell body receives constant exposure in a stationary trap.

Holding cells firmly in a flow of medium is important when using microfluidic devices, for which the factors that affect the stability of oscillating trapping need to be identified. Here, by testing different combinations of parameters, we found that the trapping stability depends on the orientation of the trap with regard to the flow direction, the ratio of scanning length and cell length, but not on scanning frequency. The most stable trapping was achieved when the l_trap_/l_cell_ ratio reached 120%, which was in accordance with a previous report stating that the trap length should be “slightly larger than the cell length”^19^. Increasing this length ratio, the trapping stability decreased, which may be due to the beam focussing at areas outside of the cell for a larger fraction of the time, so that the optical force that maintains a steady trapping is reduced.

In this work, we have found that *E. coli* can be trapped for at least 30 min without affecting viability. Being able to hold bacteria for this duration would allow for the study of dynamic processes that occur at comparable or shorter time scales. Examples are the study of gene expression (using fast-maturing fluorescent protein variants as transcriptional reporters) or protein localisation during a cycle of cell division, which can be as short as 20 min, depending on the growth medium. The shape of the FtsZ-ring, involved in cell division, has been investigated with oscillating OT^21^, but not dynamically within the same cell during its division. Another possibility would be an investigation of metabolic responses by moving cells between channels containing media of different compositions and following intracellular metabolite concentrations with FRET sensors. Moreover, when biofilm-related properties or initial surface sensing are investigated, it could be a valuable control experiment to observe cells under the microscope while they are held by OT, and thus not attached to the surface. Importantly, while optical trapping is sometimes carried out under anaerobic conditions to minimize photodamage (e.g. in refs. ^23,29^), we show that OT can also be applied to obligate aerobes. Hence, damage-free holding of single bacteria for 30 min under aerobic conditions creates many opportunities in live cell studies.

Overall, our work demonstrates that oscillating trapping is an ideal way to hold individual *E. coli* cells with strongly reduced photodamage. The correlation between cell survival and IR dose, together with the characterization of factors affecting the stability of the oscillating trap, provides a reference for the application of optical tweezers in bacterial studies. Our work shows the possibilities and limits of optical tweezer for use with live bacterial cells, and will hopefully pave the way for future use of optical tweezers in microbiology.

## Materials and Methods

### Bacterial strain and growth conditions

*Escherichia coli* K12 strain MG1655 with pP*lacZ*-*gfp* plasmid from ref. ^30^ was used in this work. M9 minimal medium^31^ with 5 g/L glucose and 25 mg/L kanamycin was used in all experiments. All solutions were prepared with MilliQ-grade water. Stock solutions of glucose (250 g/L) were adjusted to pH 7 with NaOH and filtered through a 0.2 μm PES filter. To induce expression of GFP, 500 μM IPTG was supplemented into the medium that was perfused in one channel of the microfluidic chip.

All bacterial cultures were from single colonies and were grown continuously for two days in an orbital shaker (37 °C, 300 rpm, 5 cm shaking diameter) in 50 mL medium in 500 mL flasks with silicone sponge closure. The cultures were diluted whenever approaching OD_600_ 0.5, to keep the cells in the exponential phase of growth. Once the final culture reached OD_600_ 0.4–0.5, cells were diluted to OD_600_ 0.02 with pre-warmed and aerated medium in a 50 mL tube, which was used for perfusion of cells during the experiment.

### Fabrication of microfluidic device

The microfluidic device used in this work was a polydimethylsiloxane (PDMS) slab bonded to a silanised cover glass. PDMS (Sylgard 184; Dow Corning) was poured onto a silicon wafer to mould two channels (dimensions: 430 μm (width) × 300 μm (height) × 9.7 mm (length)) and a connecting region (dimensions: 400 μm (width) × 150 μm (height) × 1.8 mm (length)) as shown in Fig. 1.

Silanisation of cover glasses was performed at room temperature. Cover glasses (22 × 40 mm, #1.5, Menzel-Gläser) were sonicated in 5 M KOH in a polytetrafluoroethylene container for 30 min, before being washed extensively with MilliQ-grade water. Then, the cover glasses were incubated with 2% (v/v) (3-aminopropyl)triethoxysilane (APTES; Sigma) in water for 2 hours. Finally, the cover glasses were sequentially washed extensively with water, acetone and water, and then dried by compressed air. Processed cover glasses were kept for up to two weeks in vacuum, as moisture in the air deteriorates the silane coating. For the bonding step, the PDMS slab and silanised cover glass were irradiated with an ozone cleaner (PSD Pro Series Digital UV Ozone System, Novascan) for 10 minutes before bonding together and baking at 120 °C for 1 hour.

### Microscope and optical tweezers setup

Microscopy was performed with a Nikon Ti-E inverted microscope, operated using NIS-Elements (AR v4.51 or v5.00, Nikon). Light sources for bright field and fluorescence imaging were a halogen lamp and a LED (pE-2, CoolLED; or AURA, Lumencor), respectively. 470 nm light was used for excitation in the GFP channel with a filter set comprising a 470/40 nm excitation filter, a 495 nm beam-splitter and a 525/50 nm emission filter (F46-470, AHF Analysentechnik). An optical tweezers system (OTM200, Thorlabs) with 1064 nm laser was used for optical trapping with a filter set comprising a 950 nm beam-splitter and a 700 nm shortpass emission filter, yielding a Gaussian beam at the focal plane. The optical tweezers were controlled by OTM software (Thorlabs), or an optical tweezers controlling module in NIS Elements v5.00 in later experiments. Trap oscillation was performed by modulating the voltage to the galvanometer-controlled mirrors in the light paths according to a linear ramp function, thus resulting in a triangle wave movement of the trap position. The overall intensity of the trapping light also followed a Gaussian distribution when oscillating.

A 100x objective (N.A. 1.45, MRD01905, Nikon) and camera (DU-897 EX, Andor) were used for image acquisition. The EM Gain Multiplier of the camera was disabled and the Readout Mode was set to 1 MHz to reduce the camera readout noise. The baseline level of camera was set to 500 at −75 °C to avoid negative intensity values. The power of the IR beams of the tweezers were measured using a power meter (PM100USB, Thorlabs) and a power sensor (S121C, Thorlabs) in the specimen plane. The Nikon Perfect Focus System was used in all time-lapse movies to ensure focus was maintained. Images in the bright field channel were focused 0.5–1.0 μm higher than those in the fluorescence channel to facilitate cell segmentation.

### Investigation of viability after IR exposure

An *E. coli* culture (OD_600_ 0.02) and glucose minimal medium containing 500 μM IPTG were driven by a pumping system (OB1, Elveflow) into the microfluidic chip via 12 cm polyether ether ketone (PEEK) flow resistors (ID: 100 μm), flow rate sensors (MFS2, Elveflow) and polytetrafluoroethylene (PTFE) tubings (ID: 0.79 mm). Prior to experiments measuring the survival percentage after optical trapping, the chip was washed with medium for at least 10 min before loading cells. During flushing, Exit 1 (Fig. 1), which was connected with thin PTFE tubing (ID: 0.30 mm) was opened for removing air in the chip and was closed once the chip was filled up. After the flushing step, to maintain high pressure in the chip, Exit 2 was connected to a 12 cm PEEK flow resistor in experiments testing the stability of oscillating traps, or a 31 cm PEEK flow resistor in all other experiments.

To facilitate the catching of single bacteria, the flow rates in both channels were first set to 0.2–0.3 μL/min. Trapped cells were kept in the stationary or oscillating trap to endure certain doses of IR exposure, as specified in the main text, and during this exposure the flow rates in the bacteria and medium channel were set to 2.0 and 2.3 μL/min (corresponding to velocities of ~260 and 300 µm/s), respectively. At the end of the exposure, the flow rates in both channels were decreased again and the cells were positioned on the silanised cover glass. To facilitate visualisation of the cell for time-lapse microscopy, oscillating trapping (trap length: 3 μm, scanning frequency: 330 Hz) was activated in the last 10 seconds of IR-exposure so that the long axis of the trapped cell was parallel with the focal plane. The trapped cell was attached to the silanised cover slip by bringing the beam focus down to the bottom of the flow channel. In experiments where the cell length was required, i.e. for determination of the ratio between trap and cell lengths, bright field images of each trapped cell were taken right after the IR exposure step. After multiple bacteria had been exposed to IR light and immobilized on the cover glass, time-lapse image acquisition was started, recording bright field and GFP images every 10 minutes.

### Measuring stability of oscillating trap

The microfluidic chip was loaded with glucose minimal medium and bacterial culture as described above. The flow rates in both channels were kept at 0.2 μL/min for trapping cells. In all experiments, the trapping power was set to 72 mW in the specimen plane. Single cells that were held in a stationary optical trap were moved to the centre of the medium channel, 0.8 mm upstream of the connecting region. Oscillation of the trap was then activated and oriented either perpendicular or parallel to the medium flow direction and an image was taken for measuring the cell length. The trapped cell was then elevated to 15 μm above the glass surface and the flow rates were set to 3 and 4 μL/min (velocities of ~390 and 520 µm/s) in the bacteria and medium channel, respectively. Here, the “rapidity” and “responsiveness” of the pumping system were both set to 0.03 to slowly increase flow rates. When the cell was flushed from the optical trap, the flow rate in the medium channel was recorded.

### Analysis of cell length, GFP expression and cell growth

To analyse cell length, size and GFP expression, bright field images from time-lapse movies were segmented into regions of interest (ROI) using MicrobeJ^32^, a plug-in for ImageJ^33^. The ROIs were then transferred to corresponding fluorescence images to obtain intracellular fluorescence intensities. Here, the GFP intensity of each cell was determined by subtracting the mean fluorescence intensity of the surrounding area with no cells from the mean fluorescence intensity in the ROI. The viability of trapped cells was assessed by means of growth rates and promoter activities. Cells were regarded alive if, within the observation time, their mean growth rate was at least 0.10 h^−1^ and the P_lacZ_ promoter activity was at least 5 RNU pixel^−1^ h^−1^ (calculated as in Ref. ^34^, using the maturation time of GFPmut2 from ref. ^35^). With these threshold values, the fluorescence intensity of the trapped cell would increase about twofold in two hours.

## Supplementary information

Supplementary figures

## Data Availability

The datasets generated during and/or analysed during the current study are available from the corresponding author on reasonable request.
