# Supplementary material for: Manipulating rod-shaped bacteria with optical tweezers
Source: Sci Rep. 2019 Dec 13;9:19086. doi: 10.1038/s41598-019-55657-y (PMC6911073; doi:10.1038/s41598-019-55657-y)
Supplement: Supplementary file 1 — Supplementary figures [file 41598_2019_55657_MOESM1_ESM.docx]

**Supplementary figures**

**Manipulating rod-shaped bacteria with optical tweezers**

Zheng Zhang^#a^, Tom E. P. Kimkes^#a^ and Matthias Heinemann*^a^

^a^ Molecular Systems Biology, Groningen Biomolecular Sciences and Biotechnology Institute, University of Groningen, Nijenborgh 4, 9747 AG Groningen, the Netherlands

^#^ These authors contributed equally to this work.

* Corresponding author: Phone: +31 50 363 8146, E-mail: m.heinemann@rug.nl


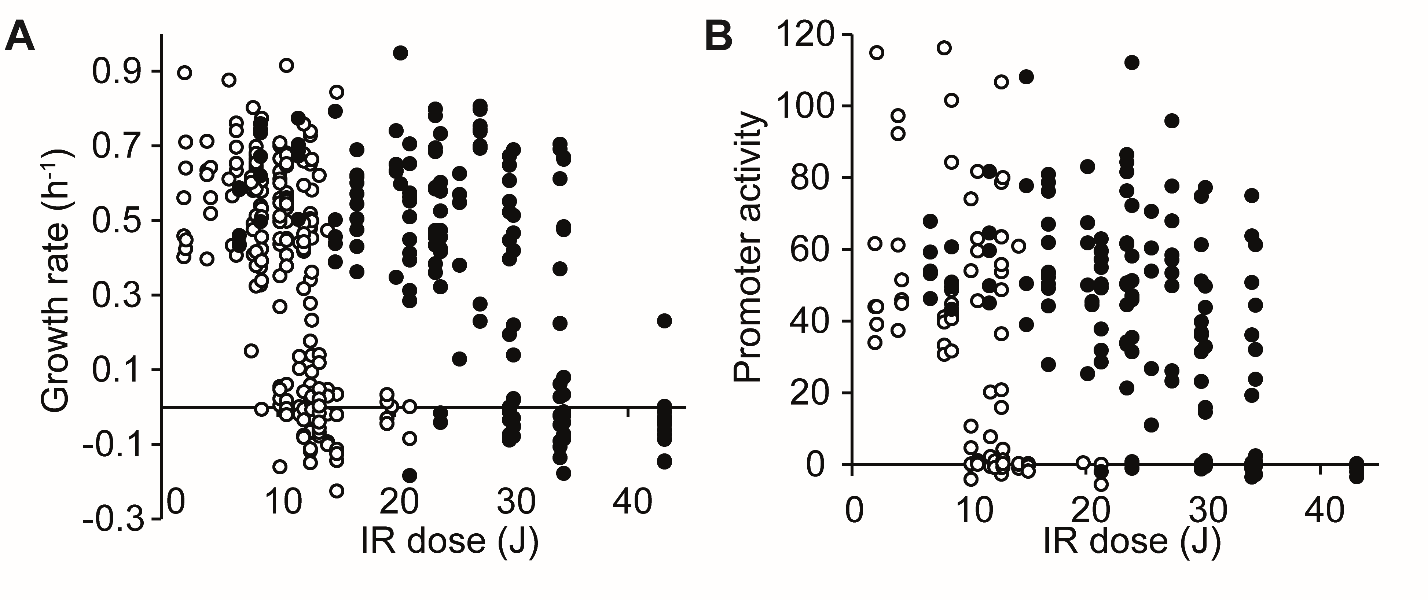


**Figure S1. Growth rate and promoter activity of optically trapped cells. (**A) The growth rate and (B) the P*_lacZ_* promoter activity (in RNU pixel^-1^ h^-1^) of optically trapped bacteria. The growth rates and promoter activities were determined from 2.5 hours after the bacteria had been positioned on the silanised cover glass. Data are from the same experiments as Figure 2A.


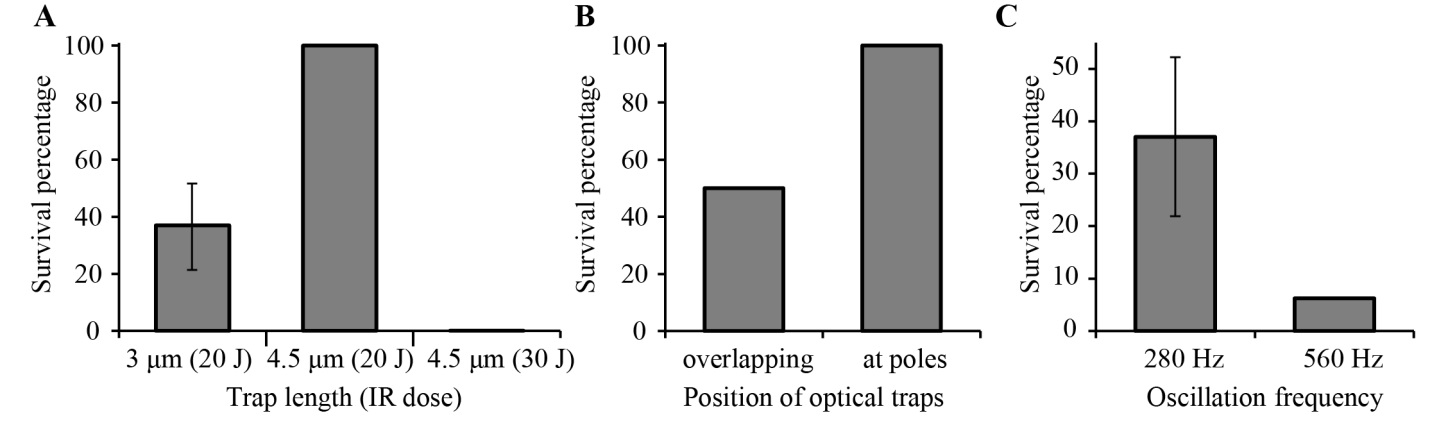


**Figure S2. Survival of trapped cells when several parameters of trapping are altered.** (A) Comparison of two oscillation lengths with and without adjustment of laser power for keeping a constant flux density. When the trap length is increased without compensation, thus distributing the IR dose over a larger area, survival is increased. However, when power compensation is applied, then longer traps with higher intensity lead to reduced survival. n = 27 for the conditions ‘3 μm, 20 J’ and ‘4.5 μm, 30 J’ in 3 independent experiments, and n = 10 for ‘4.5 μm, 20 J’. (B) Survival percentage after trapping cells with two stationary beams focused either at the same position (effectively a single stationary trap) or at the cell poles (achieving a cell orientation parallel to the focal plane). n = 16 for each condition, 2 independent experiments. (C) Survival after being held in an oscillating trap at either of two different frequencies. n = 27 for 280 Hz and n = 16 for 560 Hz.


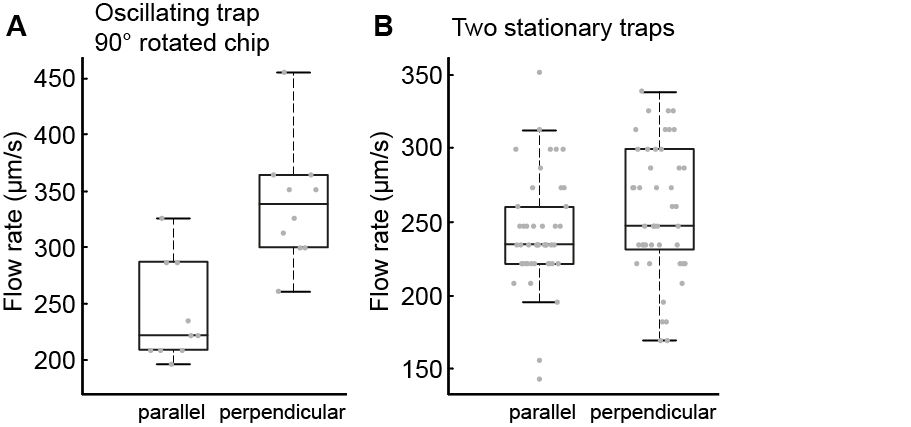


**Figure S3. Stability of parallel and perpendicular orientations.** (A) Repetition of the experiment in Figure 3, but with a 90° rotated microfluidic chip, to rule out any technical artefacts that caused the perpendicular orientation to be more stable. In the rotated channel, trapping cells perpendicularly to the flow direction is again more stable (Student t-test: p = 0.0003). n = 10 per orientation. (B) When cells are trapped with two stationary beams, there is no significant difference in stability. n = 45 per orientation, 3 independent experiments.


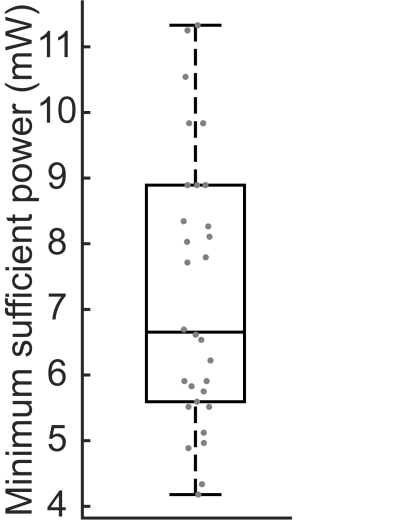


**Figure S4. Minimum IR power to retain a bacterium in a 0.5 μL/min flow rate.**  After capturing a single bacterium in an oscillating optical trap with l_trap_/l_cell_ = 1.2, oriented perpendicular to the medium flow direction, the laser power was gradually decreased until the bacterium was lost. This power was recorded as the minimum power needed to hold a cell. For 30 cells, the mean ± standard deviation was 7.2 ± 2.0 mW.
